# The use of heart rate variability, oxygen saturation, and anthropometric data with machine learning to predict the presence and severity of obstructive sleep apnea

**DOI:** 10.3389/fcvm.2025.1389402

**Published:** 2025-03-14

**Authors:** Rafael Rodrigues dos Santos, Matheo Bellini Marumo, Alan Luiz Eckeli, Helio Cesar Salgado, Luiz Eduardo Virgílio Silva, Renato Tinós, Rubens Fazan

**Affiliations:** ^1^ Department of Physiology, School of Medicine of Ribeirao Preto, University of Sao Paulo, Ribeirão Preto, Brazil; ^2^ Department of Computing and Mathematics, Faculty of Philosophy, Sciences and Letters, University of Sao Paulo, Ribeirão Preto, Brazil; ^3^ Department of Neuroscience and Behavior Sciences, Division of Neurology, School of Medicine of Ribeirao Preto, University of Sao Paulo, Ribeirão Preto, Brazil; ^4^ Department of Biomedical and Health Informatics, Children’s Hospital of Philadelphia, Philadelphia, PA, United States

**Keywords:** obstructive sleep apnea, autonomic modulation of the heart, heart rate variability, oxygen saturation, machine learning

## Abstract

**Introduction:**

Obstructive sleep apnea (OSA) is a prevalent sleep disorder with a high rate of undiagnosed patients, primarily due to the complexity of its diagnosis made by polysomnography (PSG). Considering the severe comorbidities associated with OSA, especially in the cardiovascular system, the development of early screening tools for this disease is imperative. Heart rate variability (HRV) is a simple and non-invasive approach used as a probe to evaluate cardiac autonomic modulation, with a variety of newly developed indices lacking studies with OSA patients.

**Objectives:**

We aimed to evaluate numerous HRV indices, derived from linear but mainly nonlinear indices, combined or not with oxygen saturation indices, for detecting the presence and severity of OSA using machine learning models.

**Methods:**

ECG waveforms were collected from 291 PSG recordings to calculate 34 HRV indices. Minimum oxygen saturation value during sleep (SatMin), the percentage of total sleep time the patient spent with oxygen saturation below 90% (T90), and patient anthropometric data were also considered as inputs to the models. The Apnea-Hypopnea Index (AHI) was used to categorize into severity classes of OSA (normal, mild, moderate, severe) to train multiclass or binary (normal-to-mild and moderate-to-severe) classification models, using the Random Forest (RF) algorithm. Since the OSA severity groups were unbalanced, we used the Synthetic Minority Over-sampling Technique (SMOTE) to oversample the minority classes.

**Results:**

Multiclass models achieved a mean area under the ROC curve (AUROC) of 0.92 and 0.86 in classifying normal individuals and severe OSA patients, respectively, when using all attributes. When the groups were dichotomized into normal-to-mild OSA vs. moderate-to-severe OSA, an AUROC of 0.83 was obtained. As revealed by RF, the importance of features indicates that all feature modalities (HRV, SpO_2_, and anthropometric variables) contribute to the top 10 ranks.

**Conclusion:**

The present study demonstrates the feasibility of using classification models to detect the presence and severity of OSA using these indices. Our findings have the potential to contribute to the development of rapid screening tools aimed at assisting individuals affected by this condition, to expedite diagnosis and initiate timely treatment.

## Introduction

Obstructive sleep apnea (OSA) is the most prevalent sleep disorder, characterized by repetitive events of partial and/or total obstruction of the superior airway during sleep. These obstructive events cause recurrent episodes of hypoxia and hypercapnia, leading to marked physiological disturbances (1). The gold-standard diagnostic method for OSA is polysomnography (PSG), a comprehensive exam that simultaneously records multiple physiological signals during sleep, enabling the analysis of sleep stages and their disturbances (2, 3). The diagnosis and severity assessment of OSA are determined using the Apnea-Hypopnea Index (AHI), a quantitative measure based on the number of apnea and hypopnea events per hour of total sleep time. This index is calculated by clinical specialists who analyze PSG recordings (4). Nonetheless, the time-consuming nature and associated costs of PSG tests contribute to a substantial backlog of subjects awaiting examination, leading to an increased likelihood of underdiagnosis for OSA from individual to population (5). The recent COVID-19 pandemic worsened this scenario, reducing the number of PSG exams, mainly during lockdown periods (6).

It is well determined that OSA is associated with the development of several comorbidities, especially the ones related to the cardiovascular system. On the other hand, due to poor sleeping, patients with OSA show a general decrease in the quality of life and have an increased risk of being involved in work and traffic accidents, putting their own and other lives in danger (7–9). Since the diagnostic of OSA is a bottleneck in this scenario, the development of new diagnostic techniques for OSA is of utmost relevance.

An increasingly valuable tool in screening for cardiovascular and systemic diseases is the examination of heart rate variability (HRV). It is a non-invasive approach that evaluates time series of cardiac intervals derived from the electrocardiogram and can provide insights into the autonomic nervous system's modulation of cardiac function (10). Many studies reported HRV as an important marker of cardiovascular risk, and a marked increasing number of approaches to studying HRV have been proposed in the near past (10–12). A robust body of studies has highlighted alterations in HRV indices in OSA (11, 13). However, most of these studies are limited to evaluating “traditional” HRV indices derived from conventional linear methods. Notably, there is a scarcity of literature exploring the use of more recent nonlinear approaches for HRV assessment (13). Given the complex dynamics of biological systems, techniques capable of addressing their non-stationarity, stochasticity, and nonlinear characteristics can be highly beneficial (14–16). Since HRV fluctuations exhibit nonlinear dynamics, linear methods are inherently limited in fully capturing the information contained in such signals (17). Therefore, there is a consensus that using a comprehensive set of HRV indices derived from both linear and nonlinear methods is the most effective approach for characterizing health and disease (18–20).

Another tool that has recently taken a vital role in medicine and biomedical sciences is artificial intelligence, in particular, machine learning. Machine learning models automatically identify patterns in a dataset and use them to make decisions (21–23). Machine learning can be pretty robust in leveraging big and complex data, allowing the creation of predictive models in several clinical settings, including diagnosis and treatment decisions, gene expression analysis, drug response, pharmacokinetics, and so on (24, 25). Hence, machine learning has emerged as a promising tool to assist clinicians in decision-making.

The present study aims to evaluate the utility of a comprehensive set of HRV indices in predicting the classification of individuals with suspected OSA into different severity levels (no OSA, mild OSA, moderate OSA, severe OSA) or in a binary classification based on an AHI cutoff of 15 (normal-to-mild OSA vs. moderate-to-severe OSA). To conduct this evaluation, machine learning models were trained using a comprehensive set of linear and nonlinear HRV indices, along with demographic and anthropometric variables. Additionally, we investigate how models incorporating HRV indices perform compared to models using only SpO₂ indices or a combination of both. We hypothesize that a thorough HRV profiling during sleep can function as a screening tool to identify individuals more likely to have OSA, thereby assisting in the management of the waiting list for PSG exams.

## Methods

### Data acquisition

Four hundred thirty-eight (438) PSG exams, performed between 2015 and 2022 in the University Hospital of the Ribeirao Preto Medical School from the University of Sao Paulo, were collected. All the protocol was approved by the Human Research Ethics Committee of the same hospital (Protocol: 42058720.6.000.5440/4.550.2327). The PSG exam records high-resolution waveforms such as the electroencephalogram (EEG), electrooculogram (EOG), electromyogram (EMG), electrocardiogram (ECG), thoracic and abdominal respiratory inductive plethysmography straps, pulse oximetry (SpO_2_), nasal pressure transducer system and nasal and mouth thermocouple airflow sensor to monitor the airflow, microphone to detect snores, sensor to determine body position, and a video camera to monitor the patient during sleep. The signals were exported to a European Data Format (EDF) file for further analysis.

The ECG waveforms (sampled at 512 Hz) were read from the EDF files and analyzed using the software *LabChart* (ECG module for *LabChart*, ADInstruments, Dunedin, New Zealand). Six segments of 15 min were obtained from each patient, one for each of the first 6 h of ECG recording during sleep. Segments were selected based on the quality of the ECG signal by visual inspection of the best 15 min periods of the ECG within each hour, i.e., periods with the most minor interferences from external noise, movement artifacts, arrhythmias, or gasps. Following, each segment's RR intervals (RRi) were calculated, and a series of RRi were generated for each segment. The RRi series were corrected for spurious values (e.g., beat misdetections and ectopic beats) using PyBioS software with the following procedure: for each RRi series, the baseline was estimated using a moving median window of size *W*. Upper and lower tolerance threshold lines were then created by shifting the baseline series up and down by a percentage *T* of the baseline average (26). RRi values lying below the lower or above the upper tolerance line were replaced using linear interpolation. Corrections were at most 2.5% of total estimated beats (27).

### Inclusion/exclusion criteria

The inclusion criteria for the study were patients at least 18 years old, with a minimum time of PSG recording of 6 h. The exclusion criteria were exams with corrupted files, poor ECG signal quality, RRi series with more than 2.5% spurious values, CPAP titration, or a diagnosis of central sleep apnea. After applying the inclusion and exclusion criteria, 291 out of the 438 PSG exams were eligible. A total of 147 exams were excluded due to corrupted file (*n* = 6), poor ECG quality (*n* = 75), insufficient collected time (*n* = 22), age less than 18 (*n* = 4), CPAP titration (*n* = 3), missing data in the PSG report (*n* = 37).

### Heart rate variability

Linear and nonlinear HRV indices were calculated for each patient's six corrected RRi series. As linear methods, we used both time- and frequency-domain indices (10). In the time domain, the mean of RRi, the standard deviation of normal-to-normal intervals (SDNN), and the root mean square of the successive RRi differences (RMSSD) were calculated. In the frequency domain, the spectral analysis of RRi was used. For this approach, the RRi series were resampled at 3 Hz using cubic spline interpolation and divided into segments of 512 values overlapped by 50%. Following, after the application of a Hanning window, the segments had their spectra calculated by the periodogram (Fourier transform) and were integrated into bands of very low (VLF; <0.04 Hz.), low (LF; 0.04–0.15 Hz), and high frequencies (HF; 0.15–0.4 Hz). The mean power over all 512-point segments represented the full 15 min segment. Spectral powers were presented in absolute values (“abs”) and normalized units (LFnu and HFnu). The LF/HF ratio was also calculated (28).

Several indices were used from the “family” of nonlinear methods. Detrended fluctuation analysis (DFA), which estimates the fractal (or self-similarity) scaling present on the time series, was calculated for the scaling range 5 < *n* < 15 (α1), where *n* is the window size of RRi values considered (29). Also, seven entropy measures were calculated. Entropy is generally characterized as an unpredictability/irregularity analysis of time series, with different nuances among the entropy estimators (30). In this study, we calculated sample entropy (SampEn; sequence length *m* = 2; tolerance *r* = 0.15), fuzzy entropy (FuzzyEn; sequence length *m* = 2; tolerance *r* = 0.15; fuzzy exponent *n* = 2), distribution entropy (DistEn; sequence length *m* = 3; number of bins *M* = 512), attention entropy (AttEn), dispersion entropy (DispEn; sequence length *m* = 3; number of classes *nc* = 6), phase entropy (PhaseEn; number of sectors *k* = 16), and permutation entropy (PermEn; sequence length *m* = 3; noise added to deal with equal values). All the entropy mentioned above measures had their formalism and parameters described in detail elsewhere (31–37).

Besides fractal and entropy, two symbolic dynamics analysis methods were calculated in our study. Briefly, the method Max-Min, proposed by Porta and co-workers (38, 39), split the full range of RRi values into 6 equal bins, ranging from the maximum to the minimum RRi, and each RRi value is assigned a symbol (0–5) according to the bin it belongs. Next, “words” composed of a sequence of 3 consecutive symbols are created and classified into one out of four families, namely 0V (zero variation), 1V (one variation), 2LV (two-like variation), and 2UV (two-unlike variation). The percentage of occurrence of each pattern is calculated, generating the indices Symb-0V, Symb-1V, Symb-2LV, and Symb-2UV. Another symbolic approach used is the binary method (19). This approach is similar to Max-Min, differing by how RRi values are converted into symbols. In the binary approach, each RRi is assigned a binary symbol (0 or 1), depending on the sign of the difference between the RRi and its successive RRi. Following this, “words” composed of three successive symbols are classified into one of three families, and their percentage of occurrence is calculated. This results in the generation of the indices Bin-0V, Bin-1V, and Bin-2 V. Both symbolic dynamics approaches were previously shown to be associated with the autonomic modulation of the heart and could be considered a nonlinear alternative to the spectral analysis (19, 39, 40).

Heart rate fragmentation (HRF), a recent and interesting HRV method proposed by Costa and co-workers (18, 41), was also evaluated in the present study. HRF consists of analyzing the inflection points in the RRi series, i.e., changes from HR acceleration to deceleration and vice-versa. The symbolic dynamics approach proposed for HRF assigns each RRi difference a binary symbol (1 when RRi is decreasing and −1 when RRi is increasing; the value 0 was considered when there are no differences). The transitions between two consecutive different symbols, i.e., all except 1–1, −1 to −1, and 0–0, characterize an inflection point. Then, words of 4 consecutive symbols are evaluated, and the percentage of words with zero (W0), one (W1), two (W2), or three (W3) inflection points is calculated. The overall percentage of inflection points (PIP) was also obtained. Although the HRF approach adopted here is based on a symbolic dynamics analysis (except for PIP), we did not include HRF in the symbolic dynamics methods to clearly distinguish their interpretation. The HRF was intended to evaluate the degradation of heart rate dynamics, which appears as a fragmented heart rate.

Other important HRV approaches, such as asymmetry and acceleration/deceleration (AC/DC) capacity, were also used. Asymmetry methods estimate whether the changes in the RRi series are similar when the series is time-reversed. Here, we calculated three asymmetry methods, namely Porta's, Guzik's, and Ehlers' indices. Porta's and Guzik's indices return a value of 50% for perfectly symmetric RRi series, representing the balance between positive and negative variations within the series (42, 43). In contrast, Ehlers' index is based on the skewness of RRi differences, in which values near 0 represent a symmetric (time-reversible) series (43). On the other hand, the AC/DC calculates from the RRi series the average magnitude (capacity) of the heart to accelerate and decelerate (44).

Altogether, the aforementioned methods yield 34 indices for HRV analysis. The mean values of each HRV index were obtained from each of the six hourly RRi segments.

### PSG reports

PSG reports were provided by a certified sleep medicine physician, and some scores related to the presence and severity of OSA, as well as anthropometric information, were selected. The PSG scores collected were the AHI, the minimum oxygen saturation value during sleep (SatMin), and the percentage of total sleep time the patient stayed with oxygen saturation below 90% (T90). The AHI is based on the number of apnea/hypopnea events in each hour, averaged over the entire sleep time. An obstructive apnea event can be defined as a reduction >90% of the thermistor airflow lasting at least 10 s, associated with a respiratory effort, while hypopnea is characterized by a reduction >30% of the nasal pressure airflow in a period >10 s, associated with an oxygen desaturation >3% or arousal (4). The AHI was used to classify the level of severity of OSA. Patients with an AHI between 5 and 15 were considered mild-OSA patients; between 15 and 30 moderate; and above 30 severe-OSA patients. Individuals with an AHI below 5 were considered normal (45). As anthropometric data, the patient's gender, age, height, weight, and body mass index (BMI = kg/m²) were obtained.

### Machine learning

The machine learning (ML) models were implemented in *Python* using the *sci-kit-learn* library (46). Models were trained to classify the patient's OSA class using three different input sets of features: (1) HRV indices + anthropometric data; (2) SpO_2_ indices + anthropometric data; and (3) HRV + SpO_2_ indices + anthropometric data. Both multiclass (normal, mild, moderate, severe) and binary (normal-to-mild, moderate-to-severe) classification models were evaluated, with the later utilizing an AHI cutoff of 15 (47).

The models were created using the random forest (RF) algorithm, trained with 100 trees and without a depth limit. The performance of models was assessed by a 10-fold cross-validation scheme (48). Since our dataset is unbalanced (see Table 1), we used the *Synthetic Minority Over-sampling Technique* (SMOTE) technique (49, 50) to oversample the classes with fewer samples. Synthetic data is created by SMOTE based on a set of random samples of the minority classes and their *k*-nearest neighbors (here, *k* = 5), generating the artificial data by randomly choosing a point in the linear interpolation space between them.

**Table 1:** Anthropometric and PSG report information about normal and OSA individuals in different severity classes. Sex is the number of men (%) for each group. The other variables represent the median (1st–3rd quartiles).

Variable	Normal (*n* = 47)	Mild (*n* = 63)	Moderate (*n* = 70)	Severe (*n* = 111)
Sex (men)	12 (25.5%)	27 (42.9%)	30 (42.9%)	49 (44.1%)*
Age (years)	41 (30–55)	52 (42–63)*	56.5 (38.75–65)*	56 (46–63)*
Height (m)	1.62 (1.56–1.68)	1.66 (1.57–1.7)	1.65 (1.57–1.72)	1.63 (1.58–1.73)
Weight (kg)	71.5 (60–82)	82 (74–94)*	85.5 (76.2–99.6)*	98 (79.2–115)*^,^**^,^***
BMI	26.7 (23.9–30.7)	31.04 (28.0–33.7)*	31.01 (27.5–37.4)*	34.72 (29.3–40.6)*^,^**^,^***
AHI	2.6 (1.2–3.7)	10.0 (7.5–12.7)*	21.1 (17.7–25)*^,^**	57.7 (40.8–75.9)*^,^**^,^***
T90 (%)	0.07 (0–1.30)	1.10 (0.2–9.03)*	2.75 (0.30–9.40)*	19.80 (3.5–51)*^,^**^,^***
SatMin (%)	88 (83–91)	86 (81–88)	83 (77–86)*	74 (63–82)*^,^**^,^***

AHI, apnea-hypopnea index; BMI, body mass index; SatMin, minimum oxygen saturation during sleep; T90, percentage of sleep time where oxygen saturation <90%.

* *p* < 0.05 vs. Normal.

** *p* < 0.05 vs. Normal and Mild.

*** *p* < 0.05 vs. Normal, Mild and Moderate.

To assess the importance of each feature, the feature importance assigned by the RF algorithm using the entire dataset was evaluated. RF computes feature importance by measuring the impurity decrease associated with each feature within each decision tree created in the model.

### Statistical analysis

The differences in clinical and anthropometric variables among the four groups of OSA were tested using Chi-squared (gender differences) and Kruskal–Wallis with Dunn's *post-hoc* tests (other variables). To evaluate the performance of the classification models, the area under the receiver operating characteristic curve (AUROC) was calculated. AUROC = 0.5 represents a random model, while AUROC = 1 points to a perfect classification model (20–24). The AUROC 95% confidence intervals were calculated using bootstrap with 2,000 repetitions. The difference between AUROC values was tested using the DeLong test followed by multi-comparison correction for a false positive rate (51–53).

## Results

Table 1 summarizes the characteristics of the 291 individuals included in the study. The summary of variables used as inputs for the machine learning models is presented in Table 2.

**Table 2:** Summary of features used as input attributes to create machine learning models in OSA prediction and severity.

Type of variable	Features
HRV: linear methods	MeanRR, SDNN, RMSSD; VLFabs, LFabs, HFabs, LFnu, HFnu, LF/HF
HRV: nonlinear methods	DFA-α1; SampEn, FuzzyEn, DispEn, PhaseEn, DistEn, PermEn, AttEn; Symb-0V, Symb-1V, Symb-2LV, Symb-2UV; Bin-0V, Bin-1V, Bin-2V; HRF-PIP, HRF-W0, HRF-W1, HRF-W2, HRF-W3; Porta's, Guzik's, Ehlers'; AC, DC
Oxygen Saturation	SatMin, T90
Anthropometric	Gender, Age, Height, Weight, BMI

HRV, heart rate variability; SatMin, minimum oxygen saturation during sleep; T90, percentage of sleep time where oxygen saturation <90%; BMI, body mass index. For a description of HRV features, see the text.

### Classification models

Table 3 shows the AUROC results obtained for both models. The AUROC for normal and severe classes were the best, independently of the input dataset. In contrast, the OSA moderate class always had the worst performance. The model created using all features (HRV + SpO_2_ + Antrop.) resulted in higher AUROC for normal and severe classes compared to the SpO_2_ model. For the moderate class, combining HRV and SpO_2_ showed superior AUROC compared to either HRV or SpO_2_ alone. For the mild class, however, no statistical differences were observed between the three models.

**Table 3:** AUROC (95% confidence interval) of the multiclass and binary models created from different input datasets.

Model	HRV + Anthrop.	SpO_2_ + Anthrop.	HRV + SpO_2_ + Anthrop.
Multiclass			
Normal	0.91 (0.86–0.95)	0.86 (0.79–0.92)	0.92 (0.87–0.96)*
Mild	0.74 (0.67–0.81)	0.74 (0.68–0.80)	0.78 (0.71–0.84)
Moderate	0.70 (0.63–0.77)	0.62 (0.54–0.70)	0.75 (0.68–0.81)**
Severe	0.84 (0.79–0.88)	0.80 (0.74–0.85)	0.86 (0.82–0.90)*
Binary			
	0.79 (0.74–0.84)	0.77 (0.71–0.82)	0.83 (0.78–0.88)**

HRV, heart rate variability; SpO_2_, oxygen saturation indices; AUROC, area under the ROC curve.

* *p* < 0.05 vs. SpO_2_ model.

** *p* < 0.05 vs. SpO_2_ and HRV models.

Table 4 shows the top 10 most important features for the models trained using all features available (i.e., HRV + SpO_2_ + Anthrop.) and either four or two classes. The oxygen saturation indices (SatMin and T90) were always the top 2 attributes. Anthropometric data, such as weight and BMI, were always in the top 5 attributes. Among HRV indices, VLFabs appear in the top 5 for both multiclass and binary models, while HRF-W0 and DFA- α1 were always in the top 10. Other HRV indices present in the top 10 list were Bin-0V, Bin-1V, HRF-W0, and SampEn. The attribute that contributed the most to one model was T90, reaching a maximum percentage of 8.2%.

**Table 4:** Top 10 attributes for the multiclass and binary models. Values represent the percentage contribution of each feature to the total impurity decrease [rank within the model].

Attribute	4 Classes	2 Classes
SatMin	6.7% [1]	6.4% [2]
T90	5.9% [2]	8.2% [1]
Weight	5.2% [3]	4.5% [3]
VLFabs	4.7% [4]	4.5% [4]
BMI	4% [5]	4.3% [5]
DFA-α1	2.8% [6]	2.6% [9]
Age	2.5% [7]	3.1% [8]
BIN-1V	2.5% [8]	-
HRF-W0	2.4% [9]	3.8% [6]
Height	2.4% [10]	-
SampEn	-	3.4% [7]
Bin-0V	-	2.5% [10]

## Discussion

In this study, we analyzed a set of non-invasive indices derived from different data sources for the creation of machine-learning predictive models of OSA. The combination of indices (HRV, SpO_2_) and anthropometric variables contributed to a consistent and strong performance across OSA severity classes, achieving AUROC values as high as 0.83 in the binary classification.

### Predictors of OSA

The features considered as inputs to the machine learning models were chosen based on three main factors: (1) the clinical relevance that those indices can bring related to the pathophysiology of OSA; (2) the low cost and facility to obtain the feature, particularly when compared to the PSG exam; and (3) the lack of studies reporting the predictive value of these features with OSA patients.

In OSA, personal information and anthropometric data have demonstrated clinical relevance, and many are included in risk factor calculation. It's well documented in the literature that male, obese, and older individuals have a high chance of OSA development (54). Standard oxygen saturation indices are also well studied in OSA. As hypoxia is one of the central physiological disturbances in patients with OSA, indices that a pulse oximeter can detect are clinically relevant in this population (55, 56). The assessment of these parameters has been proposed in several questionnaires and screening tools seeking to differentiate between OSA and healthy individuals (55, 57). Techniques involving ECG-derived features aiming to analyze OSA have been studied for almost 40 years, and lately, HRV has been surging as a widely studied tool in this disease (58, 59). It is already established that certain HRV indices exhibit significant differences when comparing patients with OSA to healthy subjects, likely attributed to alterations in cardiac autonomic modulation induced by OSA (60). However, most studies involving HRV and OSA have focused on traditional indices derived from the time and frequency domain, with the predictive value of most nonlinear indices being unknown in this condition (11, 13). In the present study, we demonstrated that some nonlinear HRV features are ranked in the top 10 most important for predicting OSA, although none of them rank in the top 5.

### Machine learning predictions of OSA presence and severity

Artificial intelligence models have already been used in sleep medicine, and studies aiming at building screening tests for patients with OSA using machine learning predictive models can be widely found in the literature (61). In the present study, we demonstrated an improvement in AUROC when HRV, SpO₂, and anthropometric data were combined, compared to models that used only HRV and SpO_2_ features. Some studies corroborate our findings. A recent study made by Park & Kim in a large sample of Koreans with OSA showed an AUROC of 0.69 for the RF algorithm, using linear HRV indices and anthropometric data for an AHI cutoff of 15 (62). Using HRV, SpO_2_ indices, and anthropometric data, Li and co-workers achieved an AUROC of 0.97 in differentiating OSA from normal subjects using a feed-forward neural network algorithm (63). Similarly, Zhu and co-workers used HRV and SpO_2_ indices to detect OSA and showed that the combination of HRV and SpO_2_ values had a higher performance than using HRV indices alone (64). This reinforces that the combination of these indices could be integrative for developing screening and classification models, thereby enhancing their performance.

Despite the differences between methodologies, many studies using HRV and other clinical indices to create classification models for OSA used binary OSA categorization based on different AHI cutoffs. Ravelo-Garcia and co-workers showed that the combinations of HRV and SpO_2_ indices enhanced the metrics of classifiers compared to those created with HRV indices alone, achieving an AUROC of 0.91 for an AHI cutoff of 10 (65). Baty and co-workers used the principal component analysis to select some HRV linear indices to classify OSA individuals and achieved an AUROC of 0.82 with an AHI cutoff of 18 (66). In our study, an AHI cutoff of 15 was considered because AHI > 15 is the clinical threshold established for diagnosing OSA, even when no associated symptoms are present (47).

Creating models based solely on two classes is a strategic approach, aiming to develop a screening tool focused on detecting OSA in its most severe stages. This strategy is intended to prioritize cases that would benefit from increased attention from the healthcare team. It is well-documented that patients with high AHI values can be at a higher risk of developing comorbidities (67). Moreover, severe patients remaining untreated can be at higher risk of presenting cardiovascular events in the future (68). Our best binary model provided an AUROC of 0.83, showing great potential as a screening tool for the most urgent cases, identifying the patients that could be prioritized for a complete PSG evaluation.

It's important to note that, as a retrospective study with data collected from a specialized sleep ambulatory, it was not surprising that the dataset contains a higher number of patients with OSA than normal subjects. Therefore, normal individuals composed the minority class in our group (16.15% of the sample considering an AHI < 5, and 37.8% of the sample considering an AHI < 15). Since machine learning models are sensitive to class imbalance, SMOTE technique was implemented to avoid bias, seeking to improve the models' performance and reliability. This technique has been applied to several clinical studies, including OSA, being important in trials that could have an imbalanced number of individuals, showing an improvement in classifier models for different scenarios (62, 69–71).

### Ranking of the features by their importance

The importance of the features obtained with RF revealed that SatMin, T90, weight, and BMI consistently ranked among the top 5 features. This is consistent with the clinical importance of these indices described previously. Moreover, the two SpO_2_ indices were always the two most important features in all models, consistent with the clinical importance of these variables for OSA diagnosis (55). Nevertheless, the importance assigned to SpO_2_-derived attributes (5.9%–8.2%) does not stand out compared to the importance associated with the other features (2.4%–5.2%). Notably, the list of top 10 features comprises a combination of all three types of variables, i.e., SpO_2_, HRV, and anthropometric.

Among the HRV indices, VLFabs stands out for consistently being in the top 5 list. The VLF band of the spectral analysis does not have a clear definition of its physiological meaning. Studies have attributed its value to thermoregulation, the activity of the renin-angiotensin-aldosterone system, and other humoral factors (28). A study by Francis and co-workers showed that changes in periodic breathing and oxygen desaturation can also affect the VLF component, which can be a finding in apneic patients (72). Several other studies have highlighted the significance of the VLF band in the context of OSA (73). Apneic patients exhibit higher VLF compared to normal individuals, and it has been suggested that this alteration can be reversed through therapeutic strategies for OSA (72, 74–76). The study from Baty and co-workers also demonstrated the VLF band as an essential feature in classifying patients with OSA (66). These findings suggest that VLFabs carries valuable information about the condition of OSA. Therefore, it should be considered in the development of OSA risk factors.

In addition to VLFabs, the W0 pattern of the HRF method was also present in all top 10 feature rankings. The HRF is a recent approach that requires additional investigation to further elucidate its biological meaning. HRF indices with the most inflection points (W3 and PIP) are associated with a high cardiovascular risk (18). Nevertheless, the authors who introduced HRF emphasized that the interpretation for each index may vary among different diseases (41). In OSA, W0 may be particularly affected by the cyclic variation found in the ECG (58). Studies made by Guzik and co-workers and Jiang and co-workers, employing an asymmetry approach that quantifies the length of acceleration and deceleration runs, confirm that patients with severe OSA exhibit a high number of long runs for both acceleration and deceleration (20, 77). With long sequences of acceleration and/or deceleration runs, the RRi will be changing in the same direction (up or down) most of the time, creating a high number of “fluent” patterns in HRF (W0). This particularity of OSA may explain the higher importance associated with W0 and also with VLF power of RRi spectra, all reflecting patterns of slow oscillations of heart rate.

Other HRV nonlinear indices that appear in all the top 10 lists of features include DFA-α1, SampEn, and BIN-1V. These indices are often associated with the analysis of the system's complexity, and some of them have been evaluated in studies of OSA (12, 28, 73, 78). A previous study from our research group demonstrated that HRV nonlinear indices were sensitive in detecting differences between OSA classes (particularly severe cases) and normal individuals, with significant correlations observed between these HRV indices and the AHI (79). While the physiological interpretation of these indices may not be entirely clear, they are acknowledged to offer valuable information about the organism's health status, closely tied to the concept of “physiological complexity” (15, 80). Therefore, the physiological changes induced by OSA can also be observed at a system level, as calculated by these HRV nonlinear indices, contributing to a better assessment of OSA.

### Limitations

We acknowledge important limitations in the present study. Firstly, the classes of severity of OSA exhibit an unbalanced number of samples. While the SMOTE technique aids in addressing this limitation, it generates artificial data, introducing the possibility of producing noisy instances that may not be entirely comparable to real data. Secondly, several relevant pieces of patient information were not available, including comorbidities, medication usage, and personal history (e.g., level of physical activity, smoking, alcohol consumption, race, etc.). We recognize that these factors may play a crucial role in the disease, and their impact on predictive models should be assessed in future studies. Thirdly, the selection of RRi segments was based on a visual quality assessment to avoid artifacts, without considering the sleep stage or the presence of respiratory events.

This proof-of-concept study encourages follow-up studies with data collected during the waking period, which may be more compelling in some cases. However, it is important to emphasize that, even if the models derived from electrocardiographic recordings collected during sleep do not replicate with data collected during the waking period, a Holter electrocardiographic recording, conducted overnight, is undeniably simpler, easier, and more cost-effective than a PSG. Therefore, the models obtained in the present study emerge as valuable screening tools for patients suspected of having OSA.

## Conclusion

The present study demonstrated that a comprehensive set of HRV features, combined with SpO_2_ and patient information, can be used to train highly effective predictive models for OSA classification. These models showed strong performance across different OSA severity levels, highlighting their potential as reliable diagnostic tools. Given the non-invasive nature and ease of obtaining the evaluated features, they offer a promising approach for quick and cost-effective screening of patients suspected of having OSA. This combination of HRV, SpO_2_, and anthropometric data could enable early detection and stratification of OSA severity, facilitating timely interventions and improving patient outcomes.

## Data Availability

The raw data supporting the conclusions of this article will be made available by the authors, without undue reservation.
